# Antioxidant, Antiglycation, and Hypoglycaemic Effect of* Seriphium plumosum* Crude Plant Extracts

**DOI:** 10.1155/2017/6453567

**Published:** 2017-11-13

**Authors:** Brian K. Beseni, Victor P. Bagla, Idris Njanje, Thabe M. Matsebatlela, Leseilane Mampuru, Matlou P. Mokgotho

**Affiliations:** Department of Biochemistry, Microbiology and Biotechnology, University of Limpopo, Private Bag x1106, Sovenga 0727, South Africa

## Abstract

Diabetes is a severely debilitating metabolic disorder characterised by chronic hyperglycaemia. Traditional medicinal plants provide an important avenue for the development of novel antidiabetic agents. The antidiabetic potential of the methanol, acetone, and hexane extracts of* S. plumosum* was assessed using different parameters. These included secondary metabolite quantification, hypoglycaemic, cytotoxic effects, and GLUT4 translocation augmentation on C2C12 cells. The methanol extract contained the highest amount of total phenolic and flavonoid compounds and showed enhanced antioxidant activity. The methanol extracts had the best DPPH scavenging (EC_50_ = 0.72 mg/ml) and ferric reducing powers (EC_50_ = 2.31 mg/ml). The hexane extract resulted in the highest glucose uptake activity of 35, 77% with respect to all other treatments after a 6-hour exposure period. Immunocytochemistry technique further revealed that the increased glucose utilisation may be due to increased membrane fused GLUT4 molecules in C2C12 cells. The hexane extract was also shown to upregulate the phosphorylation of p70 S6 kinase and Akt1/2. The study highlights a probable insulin-mimetic activity of the hexane extract via the augmentation of Akt1/2 phosphorylation which is involved in the GLUT4 translocation pathway. Furthermore, the study represents the first report on the cytotoxic effect, GLUT4 translocation, and glucose uptake potential of* S. plumosum*.

## 1. Introduction

The incidence and prevalence of diabetes are on an all-time high and are expected to rise drastically in the near future unless strategic countermeasures are implemented immediately [1–4]. An estimated USD4 billion was spent on diabetes healthcare in 2013 within Africa [5]. In South Africa alone, it is estimated that the cost of managing diabetes per individual annually was approximately R5 000 in 2010 but has risen to R26 743.69 in 2015 [6]. Diabetes mellitus is a multifactorial metabolic disorder characterised by chronic hyperglycaemia. Perpetual hyperglycaemia results in exasperated rates of glycation, which is defined as the spontaneous nonenzymatic reaction of reducing sugars with proteins, lipids, or nucleic acids [7]. The end result of chronic glycation is the accumulation of a heterogeneous group of biomolecules collectively termed advanced glycation end-products (AGEs) such as pentosidine, carboxymethyllysine, crossline, and pyralline [7]. Further evidence suggests that advanced glycation end-products interact with their specific plasma membrane localised receptors for AGEs (RAGE). Interaction of the AGEs with RAGE results in altered intracellular signalling, gene expression, release of proinflammatory molecules, and free radicals [7, 8]. Glycation is therefore implicated as the major underlying cause of the host of complications observed in diabetic patients such as cardiovascular disease, nephropathy, neuropathy, and retinopathy [9]. Diabetes is known to cause oxidative stress which in turn results in increased progression of its associated complications [10]. Antioxidants therefore can help reduce these ill effects by chelating the free radicals, donation of electrons, and donation of protons that stabilise these oxidative stress products that would otherwise have deleterious effects.

Some hypoglycaemic antidiabetic therapeutic agents are known to work by mediating an increasing glucose disposal within the body. GLUT4 is the major insulin responsive glucose transporter primarily expressed in adipose tissues, skeletal muscle, and cardiac muscle [11, 12]. It is found sequestrated in intracellular vesicles within the cells in which it is expressed [13]. Upon increase in blood glucose levels, insulin acts on the cell surface membrane thereby producing a cascade of events that lead to the translocation of GLUT4 from intracellular vesicle to the cell surface membrane [12]. The cascade of signal transduction events leading to the translocation and trafficking of GLUT4 molecules in response to insulin is mediated by a host of proteins collectively known as the mitogen activated protein kinases (MAPKs). Mitogen activated protein kinases (MAPKs) are a family of proteins at the heart of various important signal transduction pathways [14–16].

*Seriphium plumosum *is commonly known as the bankrupt bush or* slangbos* [17]. It is a perennial woody dwarf shrub which can grow up to 1 m high. It has characteristically intricate branched slender stems from the ground which bear the feathery greyish small leaves [17]. It is considered as an unwanted bush encroacher weed in various parts of South Africa [17] and mainly used by the indigenous people for various nonmedicinal purposes such as a broom [18]. The Basotho people use this bush to ward off bugs by placing it under their bedding and as an antidiabetic agent [18]. This study was therefore conducted to determine the toxicology profile of* S. plumosum* and its effects on GLUT4 translocation in C2C12 muscle cells. The study further assessed the antiglycation and antioxidant effects of the plant as well as the presence of various phytochemicals contained in the crude extract.

## 2. Materials and Methods

### 2.1. Plant Collection and Verification

Leaves of* S*.* plumosum* were collected from Mankweng area, Capricorn Local Municipality, Limpopo Province, South Africa. The plant was selected based on literature surveys of reports of its antidiabetic properties by traditional healers and village elders in the Limpopo Province. The plant was sampled from the same soil strata. The identity of the plant was authenticated by Dr. B Egan, a curator at the Larry Leach Herbarium, University of Limpopo (voucher specimen number UNIN 121065).

### 2.2. Plant Extract Preparation

Air-dried whole plant materials were ground into a fine powder using a domestic warring blender. Powdered plant material (1 g) was exhaustively extracted using 10 ml each of methanol, acetone, and hexane [19]. The supernatants were filtered using a Whatman No. 1 filter paper into preweighed glass vials and air-dried under a stream of cold air. The quantity of plant materials extracted was determined and stored in air-tight glass vials in the dark until use. The dry plant extracts were reconstituted in dimethylsulphoxide (DMSO) (Sigma Aldrich™, SA) for all cell based assays or in acetone for any other assay.

### 2.3. Determination of Secondary Metabolites

The presence of different plant secondary metabolites in the crude extracts was determined using various standard chemical tests (Table 1) [20].

### 2.4. Total Phenolic Content

The total phenolic content of the different extracts were determined spectrophotometrically using Folin-Ciocalteu's phenol reagent method as described [21]. Stock solutions (100 mg/ml) of each of the different extracts were prepared. Folin-Ciocalteu reagent (50 *µ*l) and distilled water (450 *µ*l) were added to each of the extracts (100 *µ*l) and left for 5 minutes in the dark at room temperature. Thereafter 7% sodium carbonate (500 *µ*l) solution was added. Distilled water was added to make a final volume of 5000 *µ*l and the mixture allowed to stand for 90 minutes in the dark at room temperature. Absorbance of the mixture in triplicate was measured at 750 nm using a spectrophotometer (Beckman Coulter-DU730). The total phenolic content was determined by linear regression from a tannic acid calibration curve standard.

### 2.5. Total Flavonoid Content

Aluminium chloride colorimetric method was used for determination of total flavonoids [22]. A stock solution (10 mg/ml) of each of the different extracts was prepared. Each of the extracts (100 *µ*l) was mixed with 10% aluminium chloride (100 *µ*l), 1 M potassium acetate (100 *µ*l), and distilled water (2800 *µ*l). The mixture was left to stand at room temperature for 30 minutes. The absorbance of the reaction mixture was measured at 415 nm in triplicate using a spectrophotometer (Beckman Coulter-DU730). The total flavonoid content was determined by linear regression from a quercetin calibration curve standard.

### 2.6. Determination of Antiglycation Activity

Antiglycation activity of the plant extracts was determined using the bovine serum albumin assay with slight modification [23]. Bovine serum albumin (Sigma Aldrich) (500 *µ*l) was incubated with glucose (400 *µ*l) and plant extracts (100 *µ*l). Phosphate buffer saline (100 *µ*l) was used as the sample control and Arbutin (100 *µ*l) (Sigma Aldrich) as the reference standard. A negative control constituting BSA (500 *µ*l), phosphate buffer saline (400 *µ*l), and plant extracts (100 *µ*l) was included. The reaction mixture was allowed to proceed at 60°C for 72 hours and terminated by addition of 10 *µ*l of 100% (w/v) trichloroacetic acid (TCA) (Sigma Aldrich). The TCA added mixture was kept at 4°C for 10 minutes and thereafter centrifuged for 4 minutes at 13000 rpm. The precipitate was redissolved in alkaline phosphate buffer saline (pH 10) and quantified for relative amount of glycated BSA, based on fluoresce intensity in 96-well plates using a microtiter-plate multimode detector (Promega-Glomax Multidetection System). The excitation and emission wavelength used were at 370 nm and 440 nm, respectively. Five concentrations of each sample were analysed in triplicate. Percentage inhibition was calculated using the formula provided below and the sample concentration required for 50% inhibition of BSA glycation was calculated:

(1) %  inhibition=OD  blank−OD  sample−OD  sample  negativeOD  blank×100.

### 2.7. Quantitative DPPH Radical Scavenging Activity Assay

The antioxidant activity of each of the different extracts was quantitatively determined spectrophotometrically using the DPPH free radical scavenging assay [24]. Equal volumes of 0.2% DPPH in methanol and different concentrations (0 *µ*g/ml to 1000 *µ*g/ml) of the extracts were incubated in the dark at room temperature for 30 minutes. The DPPH in methanol solution was used as the experimental control, L-ascorbic acid (vitamin C) as positive control, and dimethylsulfoxide (DMSO) as the negative control. The decrease in absorbance was measured at 490 nm using a microtiter-plate multimode detector (Promega-Glomax Multidetection System). The degree of discolouration indicates the scavenging potential of the extracts in terms of hydrogen donating ability. The absorbance values obtained were converted to percentage scavenging activity using the following formula:

(2) %  inhibition=A490 nm  of  blank−A490 nm  of  sample×100A490 nm  of  blank.

### 2.8. Ferric Ion Reducing Power

The ferric ion reducing power of the different extracts was determined [25]. Various concentrations (0 *µ*g/ml to 1000 *µ*g/ml) of the extracts in deionised water (100 *µ*l) were prepared. A blank was prepared without extract, while ascorbic acid was used as the reference standard. These were then mixed with phosphate buffer (250 *µ*l) (pH 7.4 and concentration 0.2 M) together with potassium ferricyanide (250 *µ*l) and incubated at 50°C for 20 minutes. After incubation, aliquots of trichloroacetic acid (250 *µ*l) were added to the mixture and centrifuged at 3000 rpm for 10 minutes. The supernatant (250 *µ*l) was mixed with distilled water (250 *µ*l) and freshly prepared ferric chloride solution (50 *µ*l). The absorbance of the samples was measured at 700 nm using a microtiter-plate multimode detector (Promega-Glomax Multidetection System). Percentage reducing power was calculated according to the following formula:

(3) Percentage  reducing  power=A700 nm  of  sample−1×100A700 nm  of  blank.

The effective concentration (EC_50_) values, which represent concentrations eliciting a 50% response, were determined by regression analysis, from linear plots of concentration of the extract against the mean percentage of the antioxidant activity from three independent experiments. A low EC_50_ value represents a more effective reducing power. Experiments were done in triplicate in three independent trials.

### 2.9. Maintenance of Cell Culture

An immortalised mouse myoblast cell line (C2C12) was used in this study (ATCC, Rockville, USA). The cells were cultured and maintained in RPMI media (Lonza, BioWhittaker®), supplemented with 10% foetal bovine serum (Hyclone, Thermo Scientific) at 37°C, in an atmosphere of 5% CO_2_ in a humidified incubator (Heracell 150i CO_2_ incubator, Thermo Scientific). The cells were differentiated by culturing in RPMI media containing 2% horse serum for 4 days.

### 2.10. Cytotoxicity Assay

The cytotoxicity of the different plant extracts on C2C12 cell line were determined using the 3-(4, 5-dimethylthiazol-2-yl)-2, 5-diphenyltetrazolium bromide (MTT) assay (Sigma Aldrich, SA) as modified by Ferrari and colleagues [26]. Experiments were done in triplicate in three independent trials. Cells were seeded at an initial cell density of 2 × 10^5^ cells/ml into 96-well cell culture plates (Nunc™, Roskilde, Denmark). The adherent cell lines were incubated overnight to allow the cells to attach. The cells were treated or not with different concentrations (0 *µ*g/ml to 1000 *µ*g/ml) of the different extracts. The untreated cells served as the experimental control. Actinomycin (Sigma Aldrich, SA) and DMSO served as positive and negative controls, respectively. The plates were incubated at 37°C for 24 hours after which MTT (10 *µ*l) was added to each well. The cells were further incubated at 37°C for 2 hours. The medium was aspirated and the cells were washed once with prewarmed PBS, pH 7.4. The insoluble purple coloured formazan formed intracellularly by the action of the mitochondrial dehydrogenase of viable cells following reaction with MTT was solubilised using DMSO (100 *µ*l). The absorbance was measured at 490 nm using a microtiter-plate multimode detector (Promega-Glomax Multidetection System). The percentage of viable cells was calculated according to the following formula:

(4) $$\begin{array}{c} Percentage\;\;viability=\frac{\left(A_{490\;nm}\text{ of}\;\;sample\times 100\right)}{\left(A_{490\;nm}\text{ of}\;\;control\right)}. \end{array}$$

### 2.11. Glucose Uptake Assay

The amount of glucose taken up by differentiated C2C12 cells was quantified using the glucose uptake kit according to the manufacturer's instructions [KAT Laboratories and Medicals (PTY) LTD]. Cells at an initial seeding density of 5 × 10^4^ were treated for 1, 3, and 24 hours in the presence or absence of the different plant extracts. Untreated cells were used as the experimental control, while insulin and DMSO were used as positive and negative controls, respectively. After treatment, the media (supernatant) (1 *µ*l) from each of the treatments, including the control, were transferred into a new 96-well flat bottomed plate and then working reagent (100 *µ*l) was added, protected from light. The mixture was incubated in the dark at 37°C for 5 minutes. Absorbance at 500 nm was immediately read using a microtiter-plate multimode detector (Promega-Glomax Multidetection System). Experiments were done in triplicate in three independent trials.

### 2.12. GLUT4 Translocation Assay

The cells were differentiated by culturing in RPMI media containing 2% horse serum for 4 days. The differentiated C2C12 cells were seeded at a density of 1 × 10^5^ cells per well in 6-well plates. The cells were treated with selected concentrations (0 *µ*g/ml and 100 *µ*g/ml) of the plant extracts for 3 hours. Insulin was used as a positive control and DMSO was used as a negative control. After treatment, the cells were washed three times with 1x phosphate-buffered saline (PBS) and fixed with 80% methanol for 15 minutes and washed three times with 1x PBS. Cells were then incubated with 4′,6-diamidino-2-phenylindole (DAPI) for 30 minutes and thereafter washed three times with 1x PBS. Cells were blocked for nonspecific binding using bovine serum albumin (1 mg/ml) (Sigma Aldrich, SA) for 30 minutes and washed three times with 1x PBS. The cells were then incubated with anti-GLUT4 primary antibody diluted 500x and thereafter washed three times with 1x PBS. The cells were then incubated with secondary antibody conjugated to FITC for 1 hour and viewed using a fluorescence microscope and overlay images were captured at 100x magnification (Nikon Ti microscope).

### 2.13. MAPK Profiling Assay

The expression of 26 mitogen activated protein kinases in the cells was determined using the human MAPK profiler assay kit according to the manufacturer's instructions (RnD Systems). Differentiated C2C12 cells at a density of 6 × 10^7^ cells/ml were seeded in 25 cm^3^ cell culture flasks and treated for 3 hours in the presence or absence of plant extract at the given concentrations (0 *µ*g/ml and 100 *µ*g/ml). Insulin (50 mI/U) was used as a positive control. The cells were immediately rinsed with PBS after which lysis buffer 6 was added. The resuspended cell lysates were then rocked gently at 2–8°C for 30 minutes. The lysates were, thereafter, centrifuged at 14,000 ×g for 5 minutes, and the supernatant was transferred into a clean Eppendorf tube. The total protein quantity was immediately determined using the BCA protein assay.

Following quantification array, buffer 5 (2 ml) was pipetted into each well of the 4-well plate where it served as a block buffer. Using flat-tip tweezers, the membranes were placed in separate wells of the 4-well plates and incubated for 1 hour on a rocking platform shaker. The samples were prepared by adding up to 400 *μ*l of each sample to separate Eppendorf tubes and adjusting the volume to 1.5 ml using array buffer 1. To each Eppendorf tube reconstituted detection antibody cocktail (20 *μ*l) was added and incubated for 1 hour. Array buffer 5 was carefully aspirated from the wells of the 4-well plate and the prepared sample/antibody mixtures were gently added and incubated overnight at 4°C on a rocking platform shaker.

The membranes were carefully removed and placed into individual plastic containers containing 1x wash buffer (20 ml) and washed 3x for 10 minutes on a rocking platform shaker. The membranes were carefully placed into each of the 4-well plates containing diluted Streptavidin-HRP (2 ml) and incubated for 30 minutes at room temperature on a rocking platform shaker. After incubation the membranes were washed 3 times with 1x wash buffer. Chemi Reagent Mix (1 ml) was added evenly onto each membrane. The membranes were then washed using TBST and the transferred proteins were detected using the Super Signal West Dura chemiluminescent substrate (Thermo Scientific, USA) and antigen antibody complex was visualised by photo-detection using the Syn-Gene Image analyser (Bio-Rad, SA).

### 2.14. Statistical Analysis

The results were obtained from three independent experiments and expressed as means ± standard deviation. The statistical significance of the results was tested using one-way Analysis of Variance (ANOVA) employing the Dunnett's Multiple Comparisons Test between the control and the different treatments within the same group. The statistical significance of the results was tested using one-way ANOVA employing the Tukey-Kramer Multiple Comparisons Test. The* p* value significance is represented as asterisk (*∗*) for* p* < 0,05, two asterisks (*∗∗*) for* p* < 0,01, and three asterisks (*∗∗∗*) for* p* < 0,001.

## 3. Results

### 3.1. Plant Material Extraction

**Figure 1:**
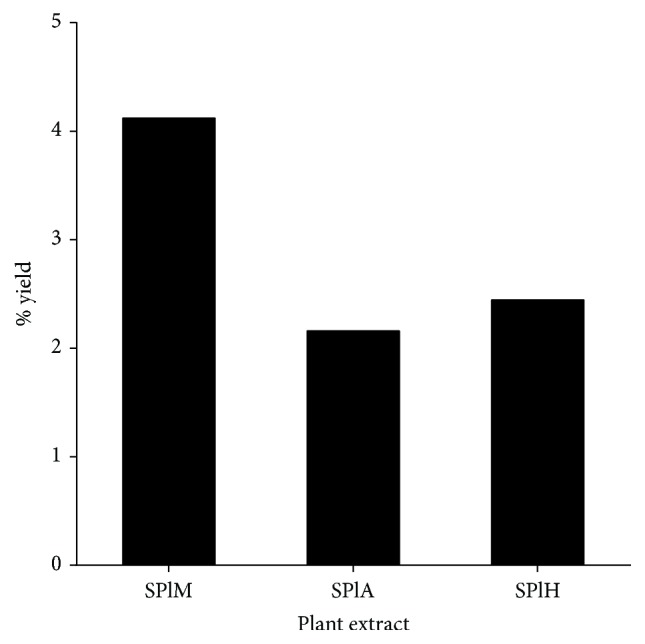
Percentage yields of the plant extracts obtained using solvents of varying polarity. SPlM:* S. plumosum* (methanol extract), SPlA:* S. plumosum* (acetone extract), and SPlH:* S. plumosum* (hexane extract).

The percentage yields of the different crude extracts obtained using solvents of varying polarity, namely, methanol, acetone, and hexane, are presented in Figure 1. Methanol had the highest extraction percentage yield of 4,12% and acetone the least (2,16%).

### 3.2. Secondary Metabolite Analysis

Qualitative analysis of the phytochemicals was performed in order to determine the presence of tannins, flavonoids, phenols, saponins, steroids, phlobatannins, glycosides, coumarins, proteins, anthraquinones, anthocyanins, leucoanthocyanins turns, and carbohydrates in all the crude plant extracts. Tannins, flavonoids, phenols, and steroids were present in all the extracts. Saponins, anthraquinones, anthocyanins, phlobatannins, glycosides, leucoanthocyanins turns, and carbohydrates were absent in all the extracts. Coumarins on the other hand were present in the methanol and acetone extracts and absent in the hexane extract (Table 2).

### 3.3. Quantitative Phenolic and Flavonoid Analysis

**Figure 2:**
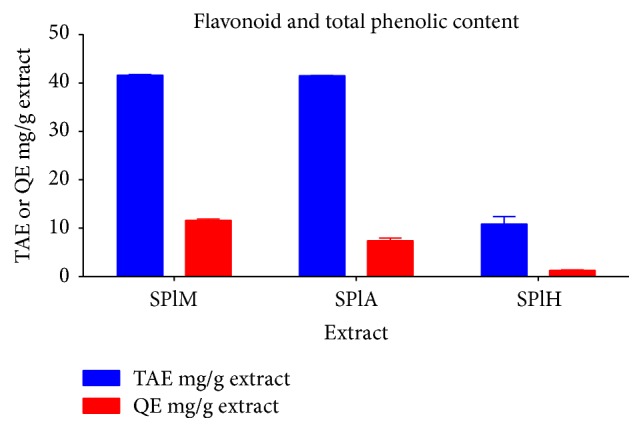
The total phenolic content of the different plant extracts represented as tannic acid equivalents (TAE mg/g) and flavonoids content in the different plant extracts represented as quercetin equivalents (QE mg/g). SPlM:* S. plumosum* (methanol extract), SPlA:* S. plumosum* (acetone extract), and SPlH:* S. plumosum* (hexane extract).

The flavonoid and total phenolic content of each of the extract were determined as quercetin and tannic acid equivalents, respectively (Figure 2), using linear regression from standard curves. The methanol and acetone extracts showed the highest amount of flavonoid and phenolic contents, while hexane extracts showed the least.

### 3.4. Quantitative FRAP and DPPH

The EC_50_ values for DPPH scavenging assay and ferric reducing power of the different plant extracts were calculated using linear regression (Table 3). The methanol extract showed the best activity among all the extracts in both assays. It exhibited the lowest EC_50_ values of 0.72 mg/ml and 2.31 mg/ml for the DPPH scavenging activity and the ferric reducing power assay, respectively. These EC_50_ values were lower than those for ascorbic acid which were 1.62 mg/ml and 3.10 mg/ml for the DPPH scavenging activity and the ferric reducing power assay, respectively (Table 3).

### 3.5. Antiglycation Activity

**Figure 3:**
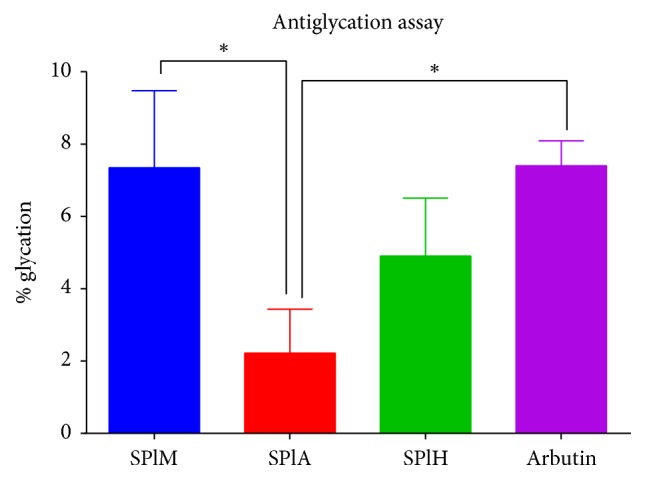
The effects of different extracts of* S. plumosum *on the glycation of bovine serum albumin (BSA). Arbutin was used as the standard reference. SPlM:* S. plumosum* (methanol extract), SPlA:* S. plumosum *(acetone extract), and SPlH:* S. plumosum* (hexane extract). The results were obtained from three independent experiments and expressed as means ± standard deviation. The statistical significance of the results was tested using one-way ANOVA employing the Tukey-Kramer Multiple Comparisons Test. The *p* value significance was represented as an asterisk (*∗*) for* p* < 0,05.

The ability of the extracts to inhibit the glycation of bovine albumin serum was conducted (Figure 3). The acetone extract exhibited the most glycation inhibitory activity among all the examined extracts, as it resulted in 2,22% glycation compared to Arbutin, a known antiglycation (7,40%) agent which was used as the positive control. On the other hand, treatment with the methanol and hexane extracts resulted in 7,30% and 4,90% glycation, respectively.

### 3.6. Cytotoxicity Analysis

**Figure 4:**
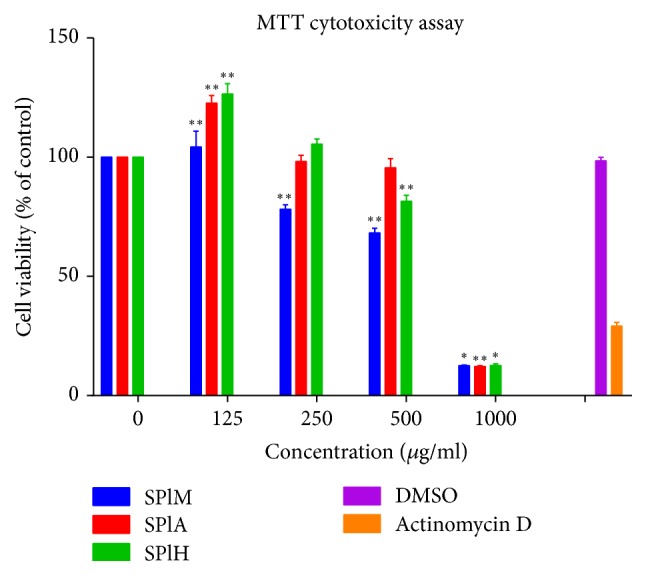
The effects of different extract concentrations of* S. plumosum *on the proliferation of murine myoblast cells (C2C12). SPlM:* S. plumosum* (methanol extract), SPlA:* S. plumosum *(acetone extract), SPlH:* S. plumosum *(hexane extract), and DMSO: dimethylsulphoxide. The results were obtained from three independent replicate experiments and expressed as means ± standard deviation. The statistical significance of the results was tested using one-way Analysis of Variance (ANOVA) employing the Dunnett's Multiple Comparisons Test. The* p* value significance was represented as an asterisk (*∗*) for* p* < 0,05 and two asterisks (*∗∗*) for* p* < 0,01.

The viability of C2C12 cell line was assessed at increasing concentrations of the different extracts using the MTT cell viability assay. The percentage cell viability was calculated relative to the untreated control. The cell viability decreased as the concentration of the various extracts increased. Actinomycin and DMSO were used as positive and negative controls, respectively. Nontoxic concentrations (125 *µ*g/mg to 500 *µ*g/mg) obtained from the assay were chosen for use in subsequent experiments. The methanol extract was shown to relatively reduce the viability of the cells as compared to the other extracts (Figure 4).

### 3.7. Glucose Uptake Assay

**Figure 5:**
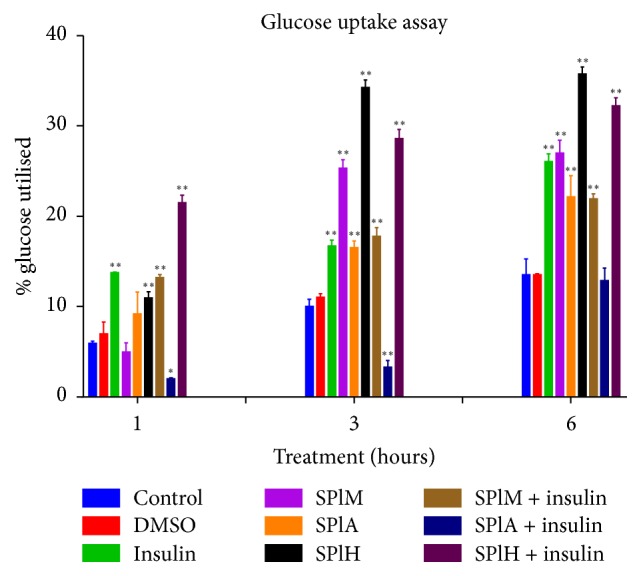
The effects of different extracts of* S. plumosum *in the presence or absence of insulin on the glucose uptake by murine myoblast cells (C2C12) at different time intervals. SPlM:* S. plumosum* (methanol extract), SPlA:* S. plumosum *(acetone extract), SPlH:* S. plumosum *(hexane extract), and DMSO: dimethylsulphoxide. The results were obtained from three independent replicate experiments and expressed as means ± standard deviation. The statistical significance of the results was tested using one-way Analysis of Variance (ANOVA) employing the Dunnett's Multiple Comparisons Test between the control and the different treatments within the same time group. The* p* value significance was represented as an asterisk (*∗*) for* p* < 0,05 and two asterisks (*∗∗*) for* p* < 0,01.

The amount of glucose utilised by the differentiated C2C12 cells exposed to different treatment conditions was quantified by the glucose uptake assay. The percentage glucose utilised was calculated with respect to the untreated control for 1, 3, and 6 hours. DMSO and insulin were used as negative and positive controls, respectively. The glucose utilisation in the DMSO treated cells was comparable with the untreated control. The combination of the plant extracts with insulin resulted in less glucose uptake as compared to the plant extract alone. This was observed for all the extracts, particularly the hexane extract, which not only resulted in the highest glucose uptake of 35,77% but was also shown to have more potent glucose uptake ability than insulin when used alone than in combination with insulin after 6 hours of exposure (32,23%). The percentage glucose utilisation is shown to increase incubation time (Figure 5).

### 3.8. Qualitative GLUT4 Translocation Assay

**Figure 6:**
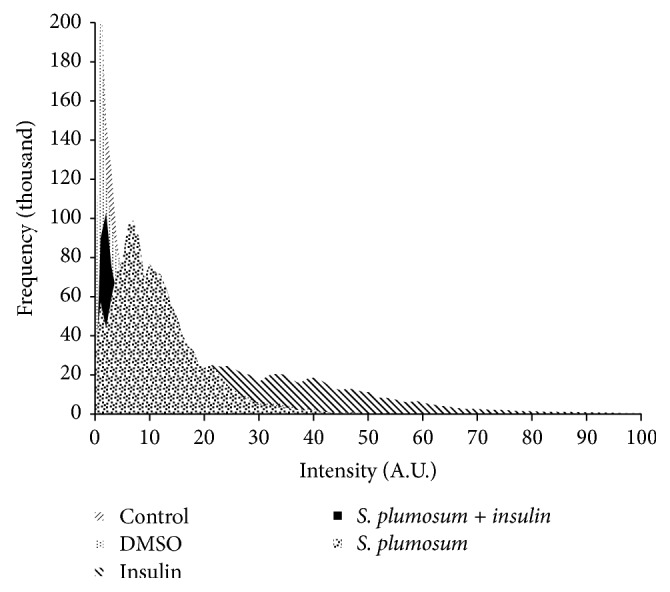
The fluorescence intensity profile of the green fluorescence from the FITC-conjugated secondary antibody which was used to stain the GLUT4 molecules of the differentiated C2C12 cells following the different depicted treatments superimposed onto each other.

The GLUT4 translocation assay was used to determine the localisation of GLUT4 molecules under various treatment conditions. The cells were treated either with or without the hexane extract (100 *µ*g/ml) since it was this extract that showed the highest glucose utilisation potential at 3 and 6 hours of exposure in the absence or presence of insulin. The untreated cells served as the negative control while DMSO and insulin served as solvent and positive controls, respectively. The cellular localisation of GLUT4 was determined by first using a primary antibody specific for GLUT4 molecule, thereafter staining with an FITC-conjugated secondary antibody to detect the areas to which the primary antibody had bound. Higher fluorescence intensity shows the translocation of GLUT4 molecules to the membrane, which indicates where the primary antibodies had greater access and bind more. On the other hand, lower green fluorescence intensities show more GLUT4 molecules sequestration in their cytoplasmic perinuclear vesicles. The fluorescence intensity profiles from an average of 4 focal points per treatment were quantified and graphed and superimposed (Figure 6). Each of the florescence intensity profile from the various treatments was then compared to that of the untreated control. DMSO, which was the solvent control, did not have an effect on the translocation of GLUT4 molecules to the membrane. The overlay of the intensity profiles following these two treatments is almost superimposable to show their similar effect on the translocation of GLUT4 molecules to the cells membrane. A shift in the fluorescence intensity profiles was observed for the cells treated with the hexane crude plant extract of* S. plumosum* both singularly and in combination with insulin as well as with insulin alone. This shift resulted in more cells with relatively higher intensities as compared to those found in the control. This means that more of the GLUT4 molecules have translocated to the cell membrane in these treatments as compared to those in the untreated control which remain mainly sequestrated in the cytoplasmic vesicles.

### 3.9. Mitogen Activated Protein Kinase Proteome Profile Analysis

**Figure 7:**
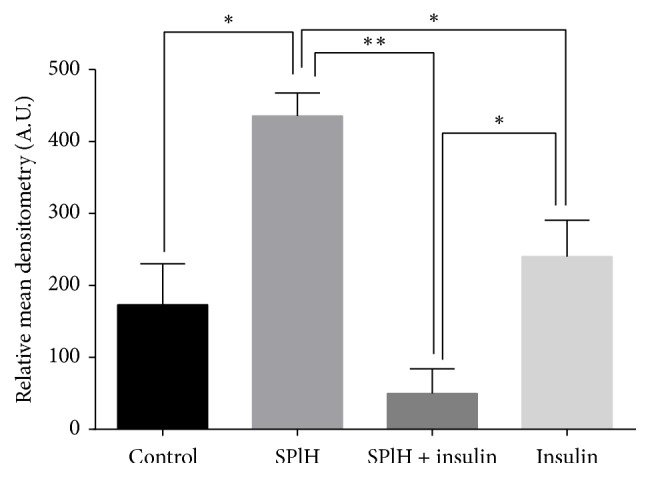
The expression of Akt1 following various treatments indicated above for a period of 3 hours. The untreated cells served as the control while insulin was the positive control. The cells were also treated with the hexane extract of* S. plumosum* only (SPlH) and in combination with insulin. The results were obtained and expressed as means ± standard deviation. The statistical significance of the results was tested using one-way ANOVA employing the Tukey-Kramer Multiple Comparisons Test. The *p* value significance was represented as an asterisk (*∗*) for* p* < 0,05 and two asterisks (*∗∗*) for* p* < 0,01.

**Figure 8:**
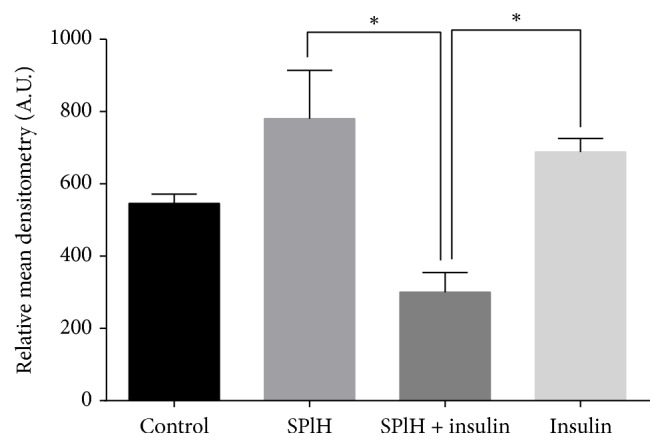
The expression of Akt2 following various treatments indicated above for a period of 3 hours. The untreated cells served as the control while insulin was the positive control. The cells were also treated with the hexane extract of* S. plumosum* only (SPlH) and in combination with insulin. The results obtained were expressed as means ± standard deviation. The statistical significance of the results was tested using one-way ANOVA employing the Tukey-Kramer Multiple Comparisons Test. The *p* value significance was represented as an asterisk (*∗*) for* p* < 0,05.

**Figure 9:**
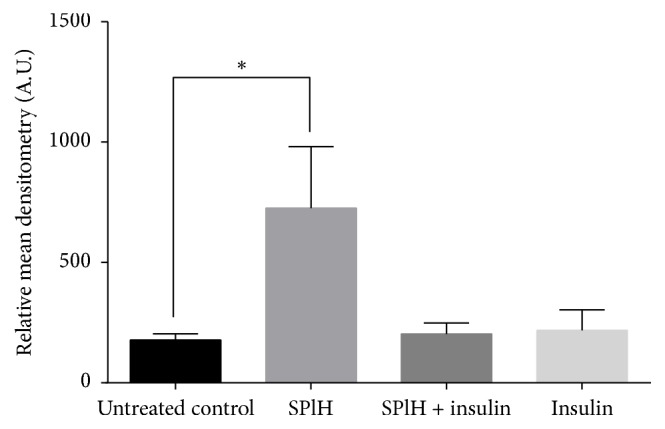
The expression of p70 S6 Kinase following various treatments indicated above for a period of 3 hours. The untreated cells served as the control while insulin was the positive control. The cells were also treated with the hexane extract of* S. plumosum* only (SPlH) and in combination with insulin. The results obtained were expressed as means ± standard deviation. The statistical significance of the results was tested using one-way ANOVA employing the Tukey-Kramer Multiple Comparisons Test. The *p* value significance was represented as an asterisk (*∗*) for* p* < 0,05.

The Human Phospho-MAPK Array was used to determine the effect of various treatments of the hexane extract on the phosphorylation of various mitogen activated protein kinases involved in the glucose uptake signal transduction pathway. Total whole cell lysates were extracted after the cells were subjected to a 3-hour treatment with the hexane extract in combination with insulin, insulin alone, and untreated cells which served as the control. The antibody-protein complexes which were observed as spots on the membranes were visualised using chemiluminescent reagents. The relative pixel densities of the spots on the membranes were quantified and the graphs were plotted for various treatments and their effects on the expression of different MAPKs that were analysed (Figures 7–9).

Expression of Akt1 was significantly higher in the cells treated with the hexane extract as compared to the control, insulin, and the combination of the hexane extract with insulin (Figure 7). Additionally, the combination of the hexane extract of* S. plumosum *and insulin resulted in significantly lower amounts of phosphorylated Akt1 as compared to insulin and the hexane extract only.

A similar trend as observed for Akt1 was also observed in the expression profile of Akt2 following different treatments (Figure 8). The hexane extract resulted in the highest expression of phosphorylated Akt2 followed by that for the positive control (insulin) and the untreated cells, respectively, whereas the combination of hexane extract and insulin resulted in the least expression of the aforementioned protein.

The ribosomal protein S6 kinase beta-1 (p70S kinase) was highly expressed in the treatment with hexane extract (Figure 9). The expression of p70S kinase was significantly higher in the cells treated with hexane extract as compared to the untreated cells.

## 4. Discussion

Plants have been employed for therapeutic purposes since ancient time. Traditional healers from across the globe practise the use of different plants and plant parts to aid in the alleviation of afflictions that result from various types of ailments. In this study methanol, acetone, and hexane were used as extraction solvents due to differences in their polarities. Methanol resulted in the highest percentage yield of 4,12%. This finding is consistent with other previous studies where methanol resulted in the highest percentage extraction yields relative to other solvents [27–29]. This finding is mainly attributed to the small molecular weight of methanol which enables it to penetrate the plant material more effectively. Although acetone resulted in the least extraction yields of 2,16%, it is of an intermediate polarity enabling it to extract compounds that are polar and nonpolar. Eloff [19] described acetone as being advantageous as an extraction solvent because of its ease of handling and it is known to extract compounds that have a broader spectrum of polarity. The qualitative phytochemical analysis conducted revealed that tannins, flavonoids, and phenols were present in all the plant extracts. These groups of phytochemicals and their derivatives were targeted in this study as they are known to be employed as templates for the manufacture of various novel therapeutic agents. The methanol extract contained slightly higher amounts of total phenolic compounds (41.63 mg/g) as compared to the acetone extracts (41.54 mg/g) while the hexane extract had the least amount (10.87 mg/g) suggesting that the majority of phenolic compounds in this plant were more polar in nature. A similar quantification profile was observed for the total flavonoids of the extracts. As flavonoids fall under the category of phenolic compounds, it was expected that the total yields of flavonoids will be less than that of the total phenolic compounds.

Polar compounds have been shown to possess higher antioxidant potential as compared to nonpolar compounds [30]. This was also observed when the antioxidant potential of the plant extracts was quantified by both the DPPH free radical assay and the ferric ion reducing power assay. The scavenging activity was observed to be in a polarity and concentration dependent manner. The methanol extract had the highest activity with an EC_50_ value of 0.72 mg/ml which was better than that of ascorbic acid which was 1.62 mg/ml. The electron donating potential of the extracts was determined by measuring their capability to reduce the ferric (Fe^3+^) ion to its ferrous (Fe^2+^) ion state. The ferric reducing power was observed to be in a polarity and concentration dependent manner. Methanol extract showed the best ferric reducing ability with an EC_50_ value of 2.31 mg/ml which was lower than that of ascorbic acid which was 3.10 mg/ml. It is likely that the phenolic compounds might also be contributing to this ability of the extracts to reduce the ferric ion to its ferrous state [31]. There has not been any report on the antioxidant potential of* S. plumosum* but several studies have shown a high degree of correlation between enhanced antioxidant activity in plants with high amounts of phenolic compounds [32, 33]. Glycation which is implicated as the major cause of the debilitating signs and symptoms in diabetic patients is a disruptive spontaneous reaction that occurs mainly between proteins and reducing sugars. The acetone extract showed the most potent BSA glycation inhibitory activity as it resulted in 2,22% glycation as compared to Arbutin which resulted in 9,73% glycation. Available reports [34] suggest plants with high concentrations of total phenolic compounds to possess high antioxidant and antiglycation activity. This report is consistent with findings in this study because the hexane extract which contained the least amount of total phenolic content also exhibited the least BSA glycation inhibitory activity. Although the precise mode by which these plants exhibit their antiglycation activity has not been established, previous study suggests that antiglycation agents may act by delaying the formation of AGEs by preventing further oxidation of Amadori product and metal-catalysed glucose oxidation [35].

Establishment of cytotoxic and noncytotoxic concentrations of different plant extracts is a crucial step in ascertaining the use and safety of plant extracts as a therapeutic agent [36]. While some plants may have therapeutic uses at lower concentrations, intake of these plants above these concentrations may be as dangerous as an overdose of western medicines. No documented report is available on the cytotoxic effect of* S. plumosum*. The methanol, acetone, and hexane extracts resulted in CC_50_ values of 518,80 *µ*g/ml, 691,03 *µ*g/ml, and 641,80 *µ*g/ml, respectively, on C2C12 cells. It was therefore concluded that since all the extracts investigated had CC_50_ values greater than 50 *µ*g/ml, they were generally noncytotoxic. A noncytotoxic concentration of 100 *µ*g/ml was determined from this analysis and used in all subsequent experiments.

Increased glucose disposal by various tissues results in a direct lowering of blood glucose in circulation. Since the hexane extract was shown to exhibit the highest glucose utilisation potential at 3 and 6 hours of exposure, it was further used in subsequent assays. Previous studies show that substances that increase glucose disposal by various peripheral organs including the muscles have hypoglycaemic effect [37]. Despite available studies [38, 39] which suggest the predominant phenolic nature of most antidiabetic compounds, the hexane extract which contained the least amount of phenolic and flavonoid content showed the best glucose utilisation effect of 35,77% which was better than that of insulin which was 26,06% after 6 hours. On the other hand this observation is in concurrence with a study by Qi and colleagues [40] which documents the antidiabetic activity of several nonphenolic phytocompounds, a report that is consistent with the present findings in this study. Treatment with the hexane extract in combination with insulin resulted in 32,23% glucose uptake after 6 hours which was lower than when the extract was used alone. This observation strongly concurs with an observation documented by Manukumar and colleagues [41] suggesting that the hexane extract acts more as an insulin mimetics rather than as an insulin sensitizer. Insulin mimetics help regulate glucose uptake by the muscle cells by producing effects that mimic that of insulin, thereby eliciting a similar cascade of reaction that results in increased glucose uptake [41]. The compounds may not necessarily bind to insulin receptor on the cell surface membrane but to any other protein within the cascade. These compounds can be helpful particularly to patients that produce relatively low amounts of insulin.

Upon an increase in blood glucose levels beyond the normal range, insulin is released by *β*-pancreatic cells. The released insulin mediates a cascade of reactions that culminate in the translocation of insulin responsive glucose transporter molecules known as GLUT4 [42, 43]. Translocation of GLUT4 molecule from its cytoplasmic vesicles to the membrane results in increased glucose transport into the cell. This is due to the fact that fusion of GLUT4 with the membrane results in increased transmembrane channels through which glucose can enter the cell [42]. The GLUT4 translocation assay was performed to investigate the shift in the distribution of GLUT4 molecules between the membrane and the cytosolic vesicle in C2C12 muscle cells following different treatments. Fluorescence intensity profiles of the different treatments against the control were used to determine these distribution patterns. The untreated cells (control) resulted in a fluorescence intensity profile that was taken to represent the basal translocation levels of GLUT4 molecules to the cell surface membrane. The fluorescence intensity profiles of the untreated cells and those treated with DMSO (negative control) were quite comparable. They both resulted in more cells with relatively low intensity and less cells with higher intensity. On the other hand, treatment with insulin alone and the hexane extract resulted in cells with higher fluorescence intensity profiles. Cells treated with the hexane extract however showed higher fluorescence intensity profile. The increase in fluorescence intensity implies that those cells had relatively more GLUT4 molecules translocating to the membranes as compared to the untreated cell. An increase in the number of GLUT4 molecules that translocated to the membrane is partly responsible for the increased glucose uptake observed in the glucose uptake assay. The effect of* S. plumosum* on both GLUT4 translocation and glucose uptake has thus far not been investigated.

The translocation of GLUT4 to the plasma membrane and the expression of GLUT1 and GLUT3 which are known to enhance glucose uptake in various insulin responsive tissues are under the control of protein kinase B (Akt) [44]. Akt is known to function downstream of phosphoinositide-3 kinase (PI_3_K) in the PI_3_K-Akt signal transduction pathway activated by insulin. In this study the hexane extract resulted in the expression of increased amounts of phosphorylated Akt1 and Akt2 with respect to the untreated control and insulin. The Akt1 isoform was phosphorylated at the Serine at position 473 while the Akt2 isoform was phosphorylated at the Serine at position 474. Phosphorylation of these different isoforms precedes their translocation into the cytoplasm where they mediate a host of functions by further phosphorylating other downstream molecules. The Akt-mediated cascade is known to mediate an increase in the translocation of GLUT4 molecules via the activation of the Akt substrate 160 (AS160) [45]. The AS160 is to date the only known Akt substrate identified that shows a phosphorylation-dependent effect on GLUT4 trafficking [45]. A study by Karlsson and colleagues [46] suggests that aberrant insulin signalling to AS160 via Akt contributes to defects in GLUT4 translocation and glucose uptake in skeletal muscle in insulin-resistant type 2 diabetic patients. Akt has since emerged as a crucial transducer of the insulin signalling cascade leading to GLUT4 translocation and glucose uptake. Akt may also be involved in activation of the nutrient-dependent Thr/Ser kinase, mTOR [47]. Activation of mTOR further results in the phosphorylation of ribosomal protein S6 kinase (p70S6K). p70 S6 kinase is a mitogen activated Ser/Thr protein kinase required for cell growth and G1 cell cycle progression [48]. In the current study the hexane extract resulted in the upregulation of phosphorylated p70S kinase which means that more energy will be directed to protein synthesis [48]. Among the proteins that are synthesised by activation of p70 S6 kinase is the GLUT4 protein. The increased amounts of p70 S6 kinase therefore may result indirectly in increased glucose uptake by both increasing the amount of GLUT4 molecules and increased energy expenditure.

## 5. Conclusion

The study reveals the antioxidant, antiglycation and hypoglycaemic potential of crude plant extracts of* S. plumosum. *Furthermore, the study documents a probable antidiabetic mode of action of the hexane extract of* S. plumosum *through enhanced glucose uptake. The increased amounts of glucose taken up by cells treated with the hexane extract were shown to result from the increase in the amount of GLUT4 molecules translocated to the cell membrane, presumably via the Akt-mediated pathway. The combination of insulin and the hexane extract of* S. plumosum* resulted in an antagonistic relationship as it resulted in lower expression of the different phosphorylated mitogen active protein kinases as compared to when the treatments were single. This may be due to the fact that the extract may contain some compounds that mask the action of insulin in the array of compounds that it contains. A plausible way of circumventing this is to isolate the pure compound in this extract that elicits the desired function which may not negatively affect the normal action of insulin. Purification and identification of compounds, which are currently under consideration, that will exhibit the observed antioxidant, antiglycation and hypoglycaemic activities may assist in the development of more potent antidiabetic pharmaceuticals.

## Figures and Tables

**Table 1:** Test for the presence of phytochemicals.

Phytoconstituent	Test	Observation
Tannins (Braymer's Test)	2 ml extract + 2 ml H_2_O + 2-3 drops of FeCl_3_ (5%)	Green precipitate
Flavonoids	1 ml extract + 1 ml Pb(OAc)4 (10%)	Yellow colouration
Phenols	2 ml extract 2 ml of 2% FeCl_3_	Blue/black colouration
Saponins	(a) 5 ml extract + 5 ml H2O + heat	Froth appears
	(b) 5 ml extract + olive oil (few drops)	Emulsion forms
Steroids (Salkowski Test)	2 ml extract + 2 ml CHCl_3_ + 2 ml H_2_SO_4_ (conc.)	Reddish brown ring at the junction
Phlobatannins (Precipitate Test)	2 ml extract + 2 ml HCl (1%) + heat	Red precipitate
Glycosides (Liebermann's Test)	2 ml extract + 2 ml CHCl_3_ + 2 ml CH_3_COOH	Violet to blue to green colouration
Coumarins	2 ml extract + 3 ml NaOH (10%)	Yellow colouration
Proteins (Xanthoproteic Test)	1 ml extract + 1 ml H_2_SO_4_ (conc.)	White precipitate
Anthraquinones (Borntrager's Test)	3 ml extract + 3 ml benzene + 5 ml NH_3_ (10%)	Pink, violet, or red colouration in ammonical layer
Anthocyanins	2 ml extract + 2 ml HCl (2N) + NH_3_	Pinkish red to bluish violet colouration
Leucoanthocyanins turns	5 ml extract + 5 ml isoamyl alcohol	Organic layer into red
Carbohydrates	2 ml extract + 2 ml iodine	A dark blue or deep purple colouration

**Table 2:** The presence/absence of various secondary metabolites in the different crude plant extracts of the different solvents.

Phytochemicals	Extracts		
	SPlM	SPlA	SPlH
Tannins	+	+	+
Flavonoids	+	+	+
Phenols	+	+	+
Saponins	−	−	−
Steroids	+	+	+
Phlobatannins	−	−	−
Glycosides	−	−	−
Coumarins	+	+	−
Proteins	−	−	−
Anthraquinones	−	−	−
Anthocyanins	−	−	−
Leucoanthocyanins turns	−	−	−
Carbohydrates	−	−	−

−: constituent absent, +: constituent present, SPlM: *Seriphium plumosum* (methanol extract), SPlA: *S. plumosum* (acetone extract), and SPlH: *S. plumosum* (hexane extract).

**Table 3:** The EC_50_ values for the DPPH scavenging assay and the ferric reducing power of the extracts.

Extract	DPPH scavenging activity	Ferric reducing power
	EC_50_ (mg/ml)	EC_50_ (mg/ml)
SPlM	0.72	2.31
SPIA	1.71	3.02
SPIH	10.58	8.06
Ascorbic acid	1.62	3.1
